# Tool Use Specific Adult Neurogenesis and Synaptogenesis in Rodent (*Octodon degus*) Hippocampus

**DOI:** 10.1371/journal.pone.0058649

**Published:** 2013-03-13

**Authors:** Noriko Kumazawa-Manita, Hiroshi Hama, Atsushi Miyawaki, Atsushi Iriki

**Affiliations:** 1 Laboratory for Symbolic Cognitive Development, RIKEN Brain Science Institute, Wako, Japan; 2 Laboratory for Cell Function Dynamics, RIKEN Brain Science Institute, Wako, Japan; Université Pierre et Marie Curie, France

## Abstract

We previously demonstrated that degus (*Octodon degus*), which are a species of small caviomorph rodents, could be trained to use a T-shaped rake as a hand tool to expand accessible spaces. To elucidate the neurobiological underpinnings of this higher brain function, we compared this tool use learning task with a simple spatial (radial maze) memory task and investigated the changes that were induced in the hippocampal neural circuits known to subserve spatial perception and learning. With the exposure to an enriched environment in home cage, adult neurogenesis in the dentate gyrus of the hippocampus was augmented by tool use learning, but not radial maze learning, when compared to control conditions. Furthermore, the proportion of new synapses formed in the CA3 region of the hippocampus, the target area for projections of mossy fiber axons emanating from newborn neurons, was specifically increased by tool use learning. Thus, active tool use behavior by rodents, learned through multiple training sessions, requires the hippocampus to generate more novel neurons and synapses than spatial information processing in radial maze learning.

## Introduction

Most basic tools are external objects that are handheld and are used to displace or reform other inanimate objects in space [1], [2]. Therefore, the cognitive capacity to use tools requires the abilities to intentionally manipulate and to flexibly reorganize spatial information that are related to the body and the environment. While this demanding mental function is considered a hallmark of human intelligence, primitive forms can occasionally be observed in non-human primates [3], [4], mammals [5], and birds [6] in the wild. Furthermore, when animals that never use tools in the wild (such as monkeys [7] and rodents [8]) are experimentally trained *de novo*, any modifications that are induced in the brain can be determined with neurobiological analyses.

New neurons are generated throughout life in the rodent hippocampal dentate gyrus, and this adult neurogenesis can be enhanced with enriched environments [9]–[11], physical activity and exercise [10], [12]–[14], spatial learning [15], and buffer stress responses and depressive behavior [16]. Exposure to enriched environments and complex social stimulations induce forms of neuronal plasticity, including long-term potentiation (LTP), in the hippocampus [17]. Therefore, neurobiological substrates that bridge the gap between spatial cognition and tool use-related voluntary manipulation of spatial information can be detected in rodent hippocampi.

Degus (*Octodon degus*) are ideal rodents for studying the neuronal substrates of tool use-related spatial information processing, as they have a developed manual dexterity that enables them to be trained to use tools [18]. In addition, degus have highly developed cognitive abilities. In this study, we compared hippocampal adult neurogenesis and synaptogenesis between tool use and spatial learning tasks that required equivalent training durations. We specifically attempted to determine if tool use-related tasks resulted in any specific changes in these hippocampal processes.

## Materials and Methods

### Animals

**Figure 1:**
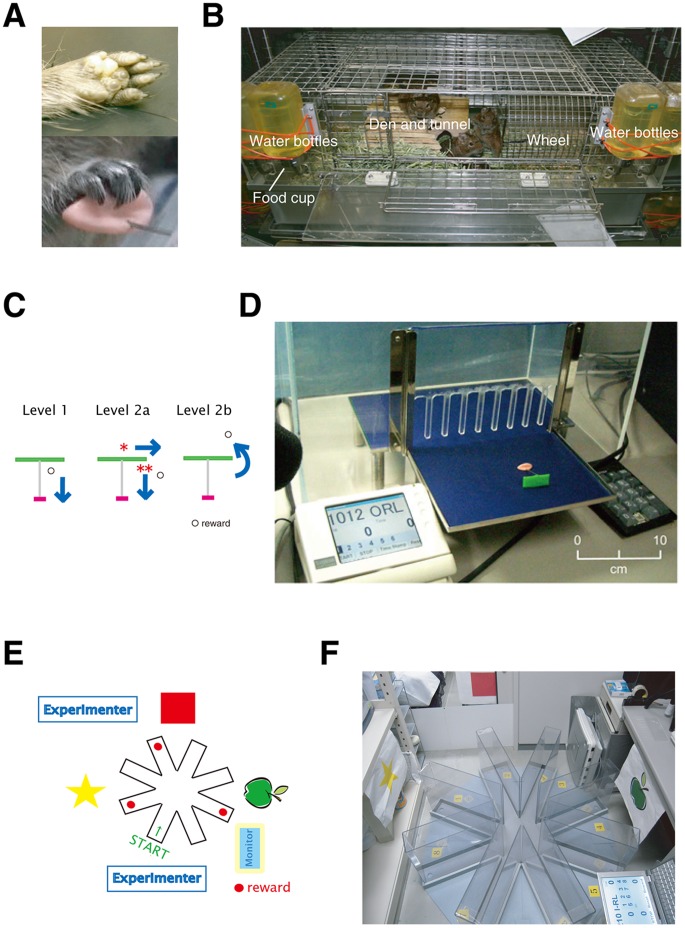
Experimental setups for behavioral analyses. A) Morphology of Degu hands. Degus have thumb-like structure (top) that allow them to manipulate tools in a dexterous fashion (bottom). B) The homecage of the degus. The degus lived in an “enriched environment” that contained a running wheel, a den, and a tunnel. C) Different levels of tool-use training processes. At Level 1, the degus simply pulled the tools (arrow) toward themselves. At Level 2, the degus had to make lateral movements (arrow*) before pulling the tool toward themselves (arrow**). At Level 2b, the degus had to place the tool beyond the reward before pulling. The degu has grabbed the tool and is about to make a lateral movement and pull the food (down). D) The experimental platform (middle), the TFT monitor for event display (left), and the numeric pad for recording (right). The scale bar is inserted as a rough reference because the photograph is tilted. The degus were placed behind the transparent fence and the experimenter sat facing them from outside of the enclosure. E) A schematic of the radial maze apparatus and the spatial cues. F) A photograph of the radial maze set-up. The experimental platform (middle), the PC for event display (right), and the spatial cues (yellow star, red square and green apple) are visible.

Adult male degus (weight 180–260 g) from five litters were used in this study. Degus, which have a similar taxonomic classification as Guinea pigs, are diurnal rodents that are native to the South American Andes Mountains in Chile. Their ecology and social systems are unique and quite different to laboratory-raised nocturnal rats and mice. Degus live in tight-knit extended family units and display complicated social behaviors, including combined maternal paternal rearing and rich repertoires of vocal communications [19]–[21]. Furthermore, since degus are diurnal, they exhibit good visual discrimination. Finally, and most importantly for the tool use task, degus have hands that contain hump-like hardened paw pads at the root that they can use as “pseudo-thumbs” that oppose their other fingers (Figure 1A, top), which allow them to manipulate tools (Figure 1A, bottom). Degus used for the present study were bred and maintained in the laboratory colony under a fixed 12-h day, 12-h night cycle at a temperature around 22°C and humidity around 50%. Three to six animals were kept in a large cage (45×70×30 cm) with a food cup and water bottles (Figure 1B). Timothy hey was provided for food and nesting materials. Food pellets, hey, and water were all provided *ad libitum*. The degus lived with their littermates (or their family) in a wooden sleeping den and tunnel with a metal running wheel. These so-called “enriched environments” were mandatory for proper maintenance of the degu colony, as previous reports showed that chronic isolation and poor environmental living conditions induces a significant decrease in the density of NADPH-disphorase-reactive neurons [22], indicating that changes in the social environment interfere with the development of limbic circuits. The degus were therefore housed in groups and in enriched environments to avoid any behavioral abnormalities, such as anxiety or offensive behaviors [23], that may have interfered with the higher cognitive functions assessed in this study.

Eight degus were used for tool use experiments (task: 4, and control: 4) and 7 were used for radial maze experiments (task: 3, control: 4). There were no differences between control and two experimental groups in genetic characteristics, hair colors and body weight (control; 212.2±4.8 g (n = 8), tool use: 216.2±16.0 g (n = 4), Radial maze: 228.7±6.2 g (n = 3), one-way ANOVA, p = 0.31) when behavioral analyses initiated at 8–9 months old. We selected these animals for the present experiments, as 1) training procedures for tool-use has been established [18], its learning processes have not much variation, so as the standard radial maze task used as a positive control, and 2) Degus produce less pups per litter than standard rodents (mice/rats) which limits numbers of age/sex-matched littermates for experiments. The experiments in this study were approved by the RIKEN Brain Science Institute animal experiment committee #H22–2B216 and complied with all institutional regulations. Before being exposed to training, each animal was habituated to the training environment by being placed in an enclosure and being given a peeled sunflower seed.

### Tool Use Training

**Figure 2:**
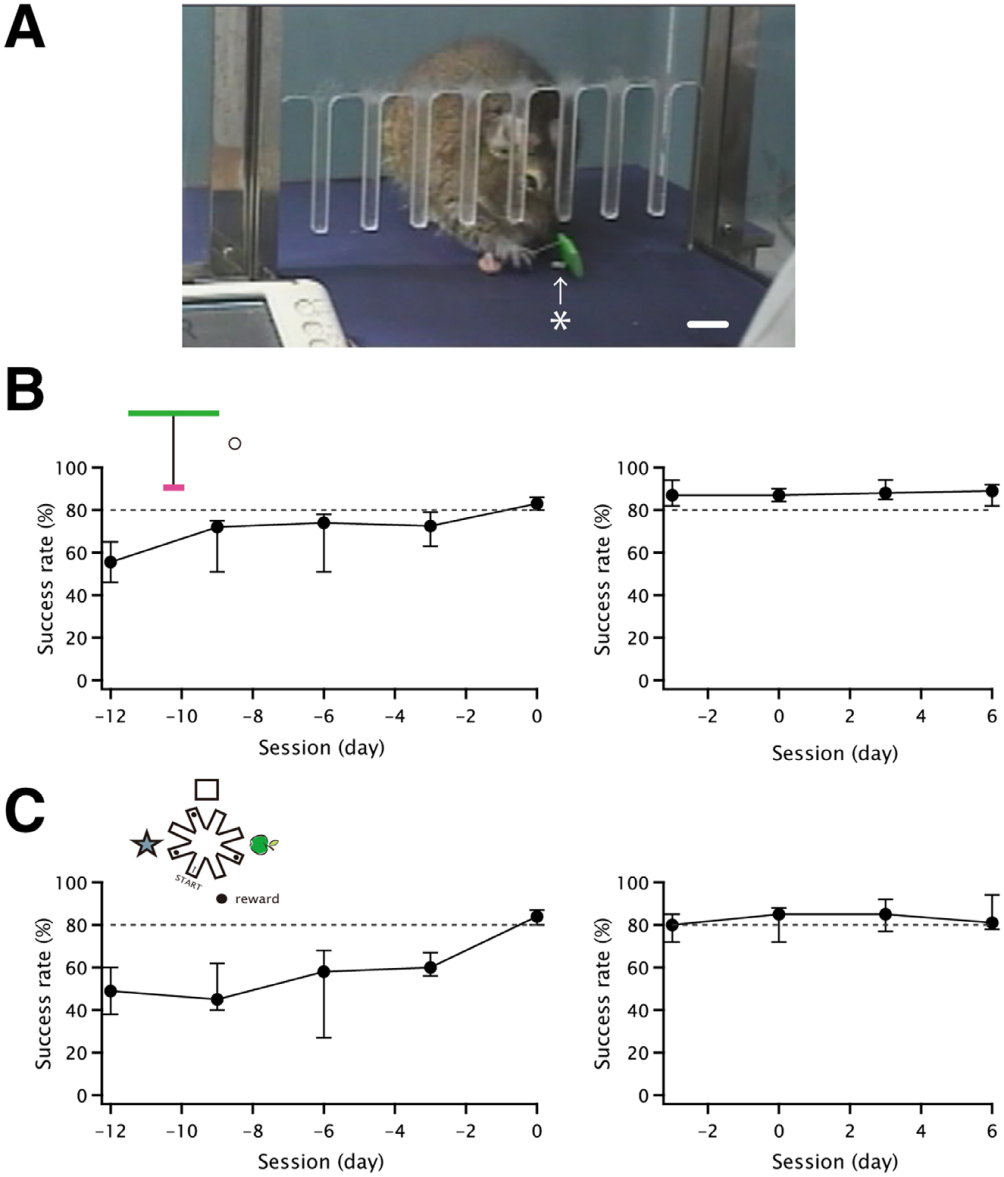
The photograph of tool use and the learning curves. A) A photograph of a degu using tool during training. The degu has grabbed the tool and is about to pull it to retrieve the food reward. An upward arrow with an asterisk indicates a food reward (a half of sunflower seed). The scale bar indicates 1 cm. B) Left: The learning curve (median ± limits of variation) observed during the tool use training phase. The success rates (ordinate) were plotted for every three sessions (abscissa) of Level 2. Sessions were numbered independently for Level 2 and were aligned at day 0 (abscissa). Each data point represents the average of four animals. Right: The success rate (median ± limits of variation) of the tool use task during the BrdU injection phase C) Left: The learning curve (median ± limits of variation) observed during the radial maze training phase. The average success rates (ordinate) were plotted for every three sessions (abscissa). Each data point represents the average of three animals. Right: The success rate (median ± limits of variation) of the radial maze task during the BrdU injection phase. For both B Left and C left, the criterion for success was an 80% or higher success rate in consecutive sessions. Day 0 is the date the criterion was satisfied. Sessions are numbered based on their alignment at the origin of the abscissa. For both B right and C right, BrdU injections started at Day 0.

Tool use training was carried out on a training platform consisted of a training stage (20×30×7 cm) and a transparent fence (45×55×0.5 cm) that separated the animal from the areas where the food rewards (peeled sunflower seeds) and tools were placed. The rake-like tools were T-shaped, with a 4 cm long wire shaft, a rectangular plane (3 cm wide×1 cm high) made of acrylic resin, and a spherical grip 1 cm in diameter. The tool weighed 2.1 g (Figure 1CD). Training sessions were monitored with two video cameras (Sony DCR-PC110) placed at the top and the upper-left side of the platform, and sessions were recorded with a camcorder (Panasonic VDR-M30). After approximately two weeks of habituation, the training sessions were performed using the successive approximation method detailed in our previous report [18]. Training was conducted at two different levels (Level 1 and 2), each of which consisted of two distinct sublevels (“a” and “b”) of difficulty based on the relative position of the tool and the reinforcement (Figure 1C). The distance between the tool and the reward was initially very close (Level 1a), but was gradually extended (Level 1b). After the animal successfully learned this task, the reward was then placed to the side of the tool so that the animal had to move the tool laterally before pulling in order to acquire the food (Level 2a). Finally, the food was placed beyond the tool, so the tool needed to be moved both forward and laterally before pulling in order to acquire the food (Level 2b). Figure 2A shows a representative example of a degu at the beginning of Level 2a of tool use training. After a reward trial, half of a sunflower seed was placed on the stage out of reach and the rake was placed within reach. On each training day, every experimental degu was put through one session that consisted of 35 to 40 trials. The criterion for acquisition was an 80% success rate for three consecutive sessions. Control animals were given the same number of food rewards, spent the same amount of time in the testing apparatuses, and the food was placed in the same locations as the tool use group. The only difference was that control animals were not given access to tools.

### Radial Maze Training

The radial maze consisted of eight equally spaced arms that radiated from an octagonal central platform. Each arm was 64.5 cm long×40 cm high×11 cm wide. A food cup of 3 cm diameter was placed at the end of each arm. Spatial cues (combination of different colors and shapes; a yellow star, a red square, and a green apple) were placed on the wall of the experimental room (Figure 1E, F). Degus are able to color discriminate between ultraviolet and visible lights [24]. The entire maze was placed on the floor. Prior to the training, the degus were habituated to the training environment by placing them in an enclosure and giving them a piece of sunflower seed. Following this habituation, the degus were placed in the maze with all of their littermates for 20 min and food rewards were scattered throughout the maze. Next, the degus were placed in the maze with three littermates for 20 min and food rewards were placed only in the food cups at the end of the arms. During the last phase of habituation, degus were placed in the maze alone for 15 min and food rewards were placed only in the food cups. After a habituation phase of approximately two weeks, the training phase was performed. Degus were trained for 4 days per week, with five trials per day. Rewards were placed at the end of three arms, and the positions of the rewards were fixed throughout the learning task (the locations of the rewards are indicated by the red circles in Figure 1E). All trials began in the middle of the same arm (green arrow, Figure 1E). Degus were trained for 4 days a week, with a session consisting of five trials per day. Degus were able to move around the maze freely. The trials ended when the degus found all of the food rewards in the maze. Trials were considered successful when a degu received all three rewards without any error entries. The criterion for acquisition was an 80% success rate for three consecutive sessions. Control animals were given the same number of food rewards, spent the same amount of time in the testing apparatuses, and the food was placed randomly in the testing apparatuses. The only difference was that control animals were able to get food rewards without remembering spatial cues. Radial maze training was performed in the same room in which tool use training was performed.

### Histological Procedures

Immediately after the acquisition of training was completed in either the tool use or radial maze task, bromodeoxyuridine (BrdU, 20 mg/kg body weight) was intraperitoneally injected for 4 consecutive days. Then, 10 days after the final injection of BrdU, animals (12–15 month old) were deeply anesthetized by pentobarbital and transcardially perfused with 4% paraformaldehyde (PFA) in phosphate buffered saline (PBS). The same procedures were applied to control animals (the numbers of days control animals were exposed to control conditions were equal to the numbers of days experimental animals were exposed to experimental conditions). After postfixation by 4% PFA in PBS and subsequent cryoprotection in 20% sucrose in PBS, coronal sections (35 µm thickness) were cut with a sliding microtome. Free-floating sections were permeabilized for 30 min in 0.1% Triton X-100 in PBS and were then subjected to immunohistochemistry. Brain sections were stained with antibodies against PSA-NCAM (a marker of immature neurons) and BrdU (a marker of newly generated cells) by an indirect immunofluorescent method. Adjacent sections were then immunostained with antibodies against PSA-NCAM and Synapsin-I (a marker of presynaptic vesicles).

### Reagents and Procedures for Immunohistochemical Reactions

We used antibodies against PSA-NCAM and BrdU to detect adult neurogenesis in the hippocampal dentate gyrus. BrdU is incorporated into the DNA of dividing cells and can then be detected by immunohistochemistry in all of the progeny of these dividing cells. PSA-NCAM is expressed during an early phase of adult neurogenesis in the dentate gyrus and is a marker of immature neurons. We also used antibodies against PSA-NCAM and Synapsin-I to detect both newly generated and exsisting synapses in the *stratum lucidum* of the CA3 region. Synapsin-I is a synaptic vesicle-associated protein [25], [26], and Synapsin-I-positive puncta that are present in PSA-NCAM-positive axons indicate newly generated synapses that originated from axons of newborn neurons [27].

To detect neurogenesis in the dentate gyrus, the primary antibodies used for immunohistochemistry were: mouse anti-polysialic acid-NCAM (PSA-NCAM) monoclonal antibody (mAb: Millipore Chemicon, 1∶400) and rat anti-BrdU mAb (abcam, 1∶300). The secondary antibodies used for immunohistochemistry were: goat anti-mouse IgM antibody Alexa Fluor 546 Conjugate (Molecular Probes) (1∶500) and goat anti-rat IgG antibody Alexa Fluor 488 Conjugate (Molecular Probes) (1∶500). For CA3 presynaptic puncta detection, the primary antibodies used for immune- histochemistry were: mouse anti-polysialic acid-NCAM (PSA-NCAM) mAb (Millipore Chemicon) (1∶400) and rabbit anti-Synapsin I pAb (Sigma) (1∶300). The secondary antibodies used for immunohistochemistry were: goat anti-rabbit IgG antibody Alexa Fluor 488 Conjugate (Molecular Probes) (1∶500) and goat anti-mouse IgM antibody Aleza Fluor 546 Conjugate (Molecular Probes) (1∶500).

### Sampling and Double-blinded Analyses Using Confocal Microscopy

#### Dentate gyrus neurogenesis

For analyses of neurogenesis in the dentate gyrus, two to three confocal images of double-positive signals in the dentate gyrus (resolution: 512×512; visual field: 250 µm square; confocal aperture: 135 µm) were acquired randomly from each slice in a blind fashion using a FV500 laser scanning microscope system (Olympus). Two to four sections were used for each animal. Ten confocal images were acquired along the z-axis (1.2 µm×12) for each visual field with an oil-immersion objective lens (PLAPO40X, numerical aperture 1.35, Olympus). Double-positive signals were counted in a blind fashion by a researcher who was not involved in acquisition of the confocal images. Proper alignment and correct image registration of both the laser lines (488 nm, argon laser and He/Ne laser 543 nm) and the detection channels were verified with double-labeled fluorescent beads (TetraSpeck Fluorescent Microscope Standards, 0.5 µm in diameter, Molecular Probes). To avoid cross detection of green and red signals, images were sequentially acquired at 488 and 543 nm, respectively. Because the corner of the dentate gyrus, where the upper and lower blades intersect, was the location where adult newborn neurons were most highly clustered, we acquired two to three images per field of the dentate gyrus to avoid wide scattering.

#### CA3 presynaptic puncta

For the analyses of presynaptic puncta in CA3, two to three confocal images of double-positive signals in the *stratum lucidum* (resolution, 1,024×1,024; visual field: 100 µm square; confocal aperture: 135 µm) were acquired randomly from each slice, in a blind fashion, using a FV500 system. Two to four slices were used for each animal. Proper alignment and correct image registration of both the laser lines and the detection channels were verified with double-labeled fluorescent beads (TetraSpeck Fluorescent Microscope Standards, 0.5 µm in diameter, Molecular Probes). Eighteen confocal images were acquired at a z-step of 0.6 µm with an Olympus FV1000 microscope equipped with an oil-immersion objective lens (PlanSApo60X, numerical aperture 1.35, Olympus) and a digital zoom of ×2.5. To avoid cross detection of green and red signals, images were sequentially acquired at 488 and 543 nm, respectively. Colocalization was quantified using Image Pro Plus 4.5.1 software (MediaCybergenetics). Synapsin I-positive puncta (Green) and PSA-NCAM-positive fiber (red) images were merged, and colocalized signals (yellow) were automatically detected in a double-blind fashion.

### Validation of Histological Procedures

In previous reports [28], [29], BrdU labeling of newborn neurons in adult animals was combined with Fluoro-Gold that was iontophoretically injected into the CA3 subfield in order to detect newly generated MF-CA3 synapses. Synapsin-I, a synaptic vesicle-associated phosphoprotein, is thought to be the leading candidate to be an activity-dependent ‘phospho switch’ that regulates synaptic vesicle tethering and release. Antibodies against Synapsin-I have been widely used to visualize presynaptic terminals in the hippocampal CA3 subfield. The immunolabeling pattern of Synapsin-I reveals large MF terminals [26] that consist of very large clusters (1–5 µm in diameter) that are densely packed with synaptic vesicles [27], [29]. Formation of presynaptic vesicle clusters is an essential step for synapse formation and the maturation process of newborn neurons [30], [31]. In this study, antibodies against Synapsin-I and PSA-NCAM were used to assess the capacity of newborn neurons to form synapses.

Visual fields were randomly selected to count adult neurogenesis (subgranular layer of dentate gyrus) and synaptogenesis (*stratum lucidum* in CA3 subfield), and no significant differences were found among slices from the same groups. When assessing the visual fields, the large Synapsin-I-positive puncta were situated directly adjacent to the apical dendrites of the pyramidal neurons, which was consistent with the expected distribution of large MF boutons in *vivo*. Because the large puncta were invariably located in the hilus and the *stratum lucidum* of the CA3 subfield, but never in the CA1 subfield [32], random acquisitions of images from the *stratum lucidum* were used to validate data.

## Results

A total of 15 degus were used in this study. This number included degus exposed to tool use (n = 4) and radial maze (n = 3) task trainings, as well as control groups (n = 4 for tool use, n = 4 for maze). The procedure that was used for tool use training was reported in our previous study [18]. Figure 2A shows a photograph of a degu using the tool. The degu has grabbed the tool and manipulate it to retrieve the reward (arrow/asterisk, a half of sunflower seed). Figure 2B illustrates the learning curve (shown as median ± limits of variation) for Level 2a tool use behavior, which is critical to the whole tool use learning processes and revealed a degree of difficulty equivalent to the radial maze task – more difficult level 2b was not preferred in this study as its time course of acquisition tended to vary across animals and require much more dates compared to the radial maze task. The learning curves of each animal were aligned, and then averaged at the time point when animals reached the criteria for acquisition of tool use learning (80% success rate for three consecutive trials). For comparison, a straightforward spatial learning task that had a similar learning curve was designed. This task consisted of an eight-beamed radial maze with baits placed at the end of three beams. Figure 2C illustrates the learning curve (median ± limits of variation, n = 3) aligned at the time point when the animals reached the criteria for the acquisition of the spatial learning (80% success rate for three consecutive sessions where rewards were obtained without error entries). For both learning curves, the average sessions, beginning 12 days prior to the completion of learning, are shown (Figure 2BC, left). A post-hoc analysis (*t*-test) confirmed that there were no significant differences between these two learning curves (P = 0.16).

BrdU was injected into the animals subjected to training once per day for 4 days after task acquisition (20 mg/kg body weight, i.p.). These animals were then fixed 10 days after the final BrdU injections (Figure 2AB, right). In the right panels of Figures 2AB, BrdU injections occurred at day “0″, and both the tool use and radial maze trainings continued throughout the 14 days shown (trainings were performed 4 days per week). The success rates of the tool use and radial maze tasks were greater than 80% both before and after the BrdU injections. The control animals were exposed to the same number of training days, but with control conditions, and were given the same amount of food in the same experimental apparatuses as the trained animals. Similar to the trained animals, control animals were fixed 14 days after the initial BrdU injections. Instead of using idle naïve controls, as is done in many other rodent experiments employed to detect enhanced neurogenesis in various learning paradigms [17] or enriched environments [9], we employed enriched controls conditions because of the characteristic nature of degu habitats. Unlike other laboratory rodents, such as rats or mice, degus require group housing in a somewhat enriched environment in order to maintain their basic physical conditions. Degus subjected to isolation in poor environments, such as living alone in standard cages, develop severe physical problems that may result in death [33]. Therefore, it was difficult to normalize naïve controls.

**Figure 3:**
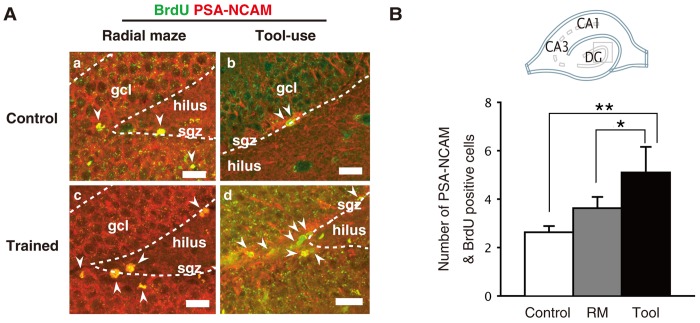
Tool use learning, but not radial maze learning, enhanced adult neurogenesis in the dentate gyrus. A) Confocal images of BrdU+/PSA-NCAM+ immunoreactivity in sections from animals sacrificed 10 days after the last BrdU injection. Arrowheads indicate nuclei that are double-labeled for BrdU (green) and PSA-NCAM (red) in the subgranular zone of the dentate gyrus. gcl: granular cell layer; sgz: subgranular zone. B) Effect of tool use or radial maze on adult neurogenesis in the dentate gyrus. The numbers of BrdU^+^/PSA-NCAM^+^ cells in the dentate gyrus (n = 4–6 per group) were plotted on the y-axis. A schematic of the hippocampus is shown in B (*top*). The images in A (square) were acquired in the subgranular zone of dentate gyrus. Asterisks refer to statistical significance: *p = 0.006, **p<0.001. The scale bars indicate 50 µm.

Five brain sections were randomly picked from 50 total sections that covered the entire dentate gyrus, and were subjected to double-blind confocal microscopic analyses to detect adult neurogenesis by double-labeling of BrdU and Polysialic acid-neural cell adhesion molecule (PSA-NCAM), BrdU is a marker of newly-generated cells and PSA-NCAM is a marker of immature neurons [34]. In all four (two experimental and two control) groups of animals, the PSA-NCAM/BrdU double-positive signals (as indicated by the arrowheads in Figure 3A) were detected in the subgranular zone (sgz, dotted line) close to the border of the granular cell layer (gcl) and adjacent to the hilus. This area is known to be a region that contains prominent adult neurogenesis [35]. There were no significant differences in the numbers of newborn cells detected between the two control groups [for Radial maze: 2.78±0.24 (n = 27 sections, N = 4 animals); and for tool use: 2.27±0.26 (n = 11 sections, N = 4 animals); one-way ANOVA, p = 0.37], indicating that the enrichment experiences between the two experimental conditions were well-controlled. Compared to the combined average of the control groups [2.63±0.24 (n = 38 sections, N = 8 animals)], the number of PSA-NCAM/BrdU double-positive neurons were significantly increased in the tool use trained group [6.31±1.11 (n = 13, N = 4)] (one-way ANOVA, ** p<0.001), but not in the radial maze trained group [3.63±0.46 (n = 16, N = 3)] (Figure 3B; one-way ANOVA, p = 0.30). In addition, the number of double-positive neurons was significantly greater in the tool use trained group than in the radial maze trained group (one-way ANOVA, * p = 0.006).

**Figure 4:**
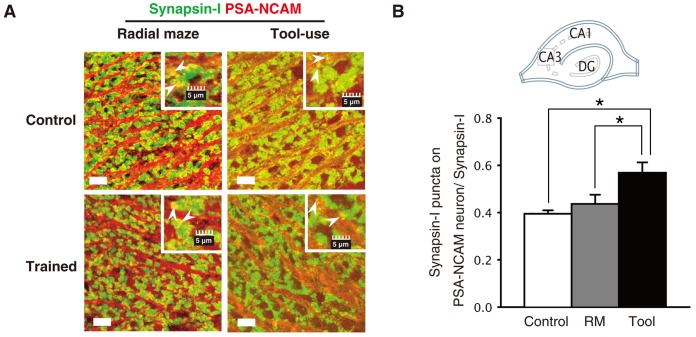
Tool use learning, but not radial maze learning, promoted maturation of newly generated neurons. A) Confocal images showing double-labeling for Synapsin-I (green) and PSA-NCAM (red) in the CA3 subfield of the hippocampus. Low magnification images are shown with high magnification insets. Representative PSA-NCAM^+^/Synapsin-I^+^ puncta are indicated by arrowheads in the inset images. B) Effects of tool use or radial maze tasks on formation of clusters of presynaptic vesicles in the CA3 subfield. The numbers of PSA-NCAM^+^/Synapsin-I^+^ puncta in the CA3 subfield were plotted along the y-axis. A schematic of the hippocampus is shown in B (*top*). Target areas (square) were randomly selected and the images were acquired in the *stratum lucidum* of the CA3 subfield. Asterisks refer to statistical significance: *p<0.01. Scale bars indicate 50 µm in the low magnification images and 5 µm in the high magnification images.

Hippocampal adult neurogenesis alters network functions through the continuous addition of new neuronal units to the network. These new neurons extend long axonal projects along the mossy fiber pathways that innervate the target CA3 pyramidal cell layer and make new synapses with dendrites of pyramidal cells that are located in the *stratum lucidum* [11], [36]. In order to determine if the tool use-induced newborn dentate neurons also had a greater capacity to form new synapses in the CA3 subfield, immunostaining with antibodies against Synapsin-I and polysialylated NCAM (PSA-NCAM) was performed on randomly selected CA3 sections (5 out of 50 total sections that covered the entire CA3 subfield) containing the primary mossy fiber tract *stratum lucidum*. Synapsin-I is a protein associated with small synaptic vesicles [37]–[39] and PSA-NCAM is a marker of immature neurons [34]. The immunostaining revealed that clusters of Synapsin-I-positive puncta (green) were colocalized with PSA-NCAM-positive mossy fibers (red) in the *stratum lucidum*. A representative image is shown in Figure 4A (arrowheads). In this image, mossy fiber terminals (1–5 µm in diameter) with densely packed synaptic vesicles can be observed [30]. To determine the probabilities of new synapse-formation, we assessed the ratios of double-labeled signals to total single Synapsin-I-positive signals [36]. The ratios of control, tool use trained, and radial maze-trained animals were 0.38±0.02 (n = 56, N = 8), 0.54±0.04 (n = 24, N = 4), and 0.40±0.04 (n = 24, N = 3), respectively (Figure 4B). The probability of new synapse formation was significantly higher in the tool use trained group than the other groups (one-way ANOVA, * p<0.01).

## Discussion

The hippocampal dentate gyrus is known to be a location where new neurons are generated throughout adulthood. These new neurons are sparsely integrated throughout the granular cell layer, and their numbers are enhanced by exposure to an enriched environment [9]. More specifically, the number of materials that are scattered throughout an enriched environment correlate with the enhancement in neurogenesis, thereby indicating that new neurons may be necessary for the understanding of complex spatial structures. In this study, the control conditions that were used provided an enriched environment, due to the ecological requirements of degus as indicated above [19]. This enriched control environment could explain why the straightforward radial maze spatial learning task did not induce enhancement in adult neurogenesis, unlike previous reports where enhanced adult neurogenesis was induced by spatial learning tasks or enriched environment when compared with idle naïve conditions [40]. Thus, the perceptual demand that was required for the radial maze task may not have exceeded the demand for the enriched control conditions. The numbers of elements that needed to be learned, and the difficulties of learning the spatial relations of the elements may not have been different enough between radial maze task and the control conditions. Regardless, the data indicate that there are elements of adult neurogenesis that are specific to tool use learning.

A previous report showed that reduction of adult neurogenesis by toxin affect fear conditioning paradigm but did not contextual fear conditioning or spatial navigation learning in Morris water maze [41]. They discussed that new born neuron are involved in the acquisition of memories which are dependent on the structure and, that all type of hippocampal-dependent learning involve new neurons for acquisition but spatial navigation learning can still occur with a very small percentage of new neurons [41].

What are the differences between radial maze and tool use learning? There are three major components of tool use learning that may be more demanding than radial maze learning and may require additional hippocampal circuit resources. One difference would be that tool use learning requires quantitatively greater numbers of items and spatial structures to be learned. For example, the locations of the beams and food need to be learned in the radial maze task, while the tools, baits, hand movements, and variations in different situations need to be learned in the tool use task. A second and more critical qualitative difference exists between the two tasks. In the tool use task, spatial structures change dynamically in time. When a tool is used to move food, then the locations of the items in space are changed and the neuronal circuitry needs to encode the new spatial locations in order to predict the future spatial relationships of the items [42], [43]. Finally, perhaps the greatest difference between the two tasks is that radial maze learning only requires “passive” perceptions of existing situations, while tool use learning requires understanding of the relationship between self and spatial structures and the ability to “actively” modify these relationships [44]. All three of these differences between the tasks indicate that tool use learning places greater demands on the neural circuitry to process information than radial maze learning.

The present study demonstrated that tool use learning induces adult neurogenesis in the hippocampal dentate gyrus of dexterous rodents (degus). These newborn neurons create novel hippocampal neural circuits by projecting their axons via the mossy fiber tract to the CA3 subfield and by forming new synapses in this target region. These enhancements in adult neurogenesis and synaptogenesis in the hippocampus may be necessary to accommodate the greater information processing demands placed on the circuitry by tool use learning relative to simple spatial learning, and also to accommodate the unique relationships between self and spatial structures that change over time. The results indicate that the central nervous system can adapt to new requirements that emerge from physical conditions (degus have peculiar hands that enable tool use), and that these adaptations may be dependent on the enhancements of existing neural resources. The data presented here may ultimately be relevant for understanding much higher human intelligence and cognitive functions.
